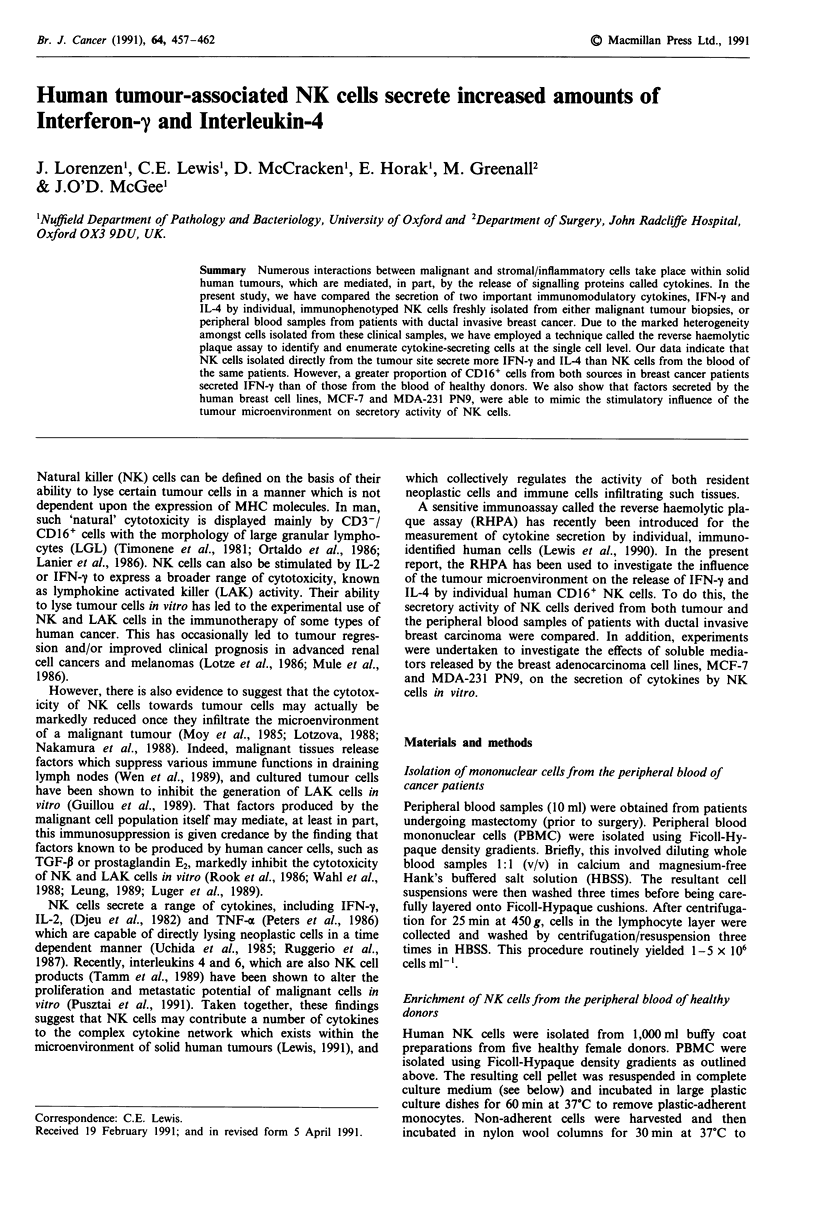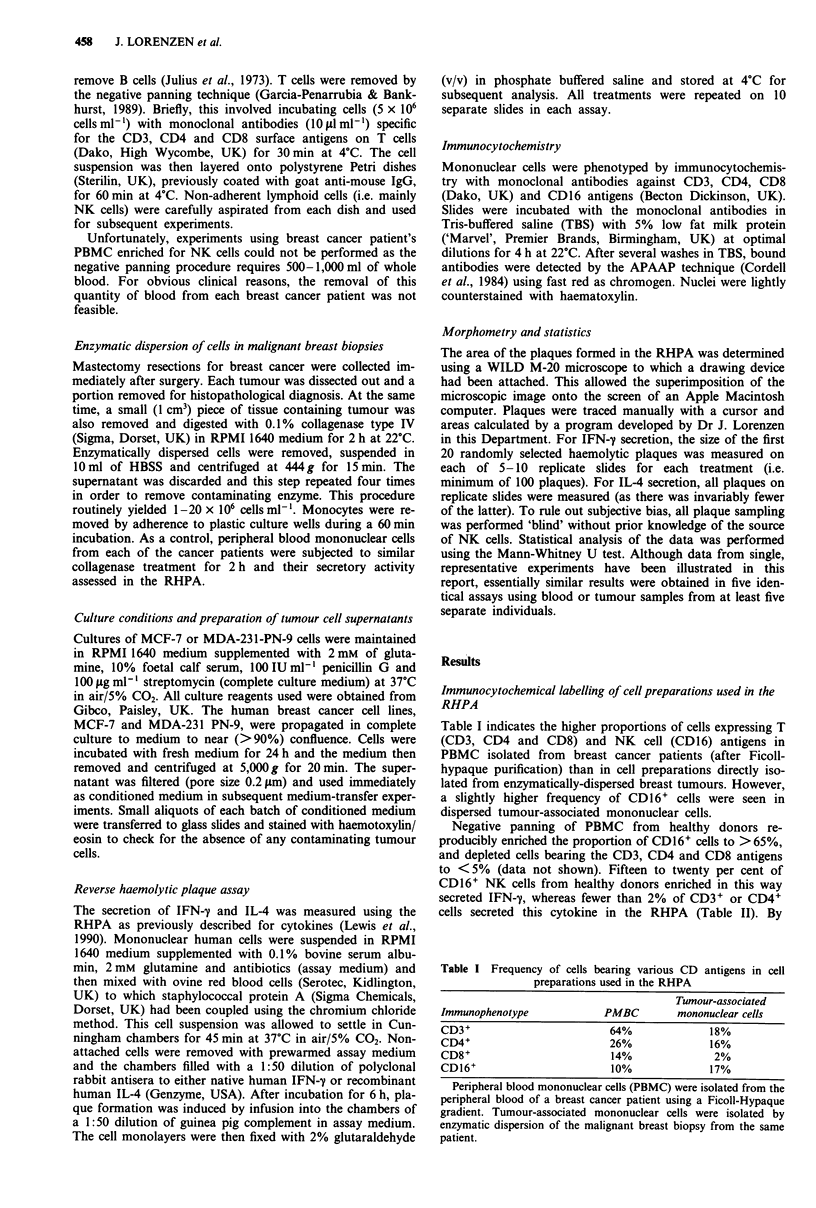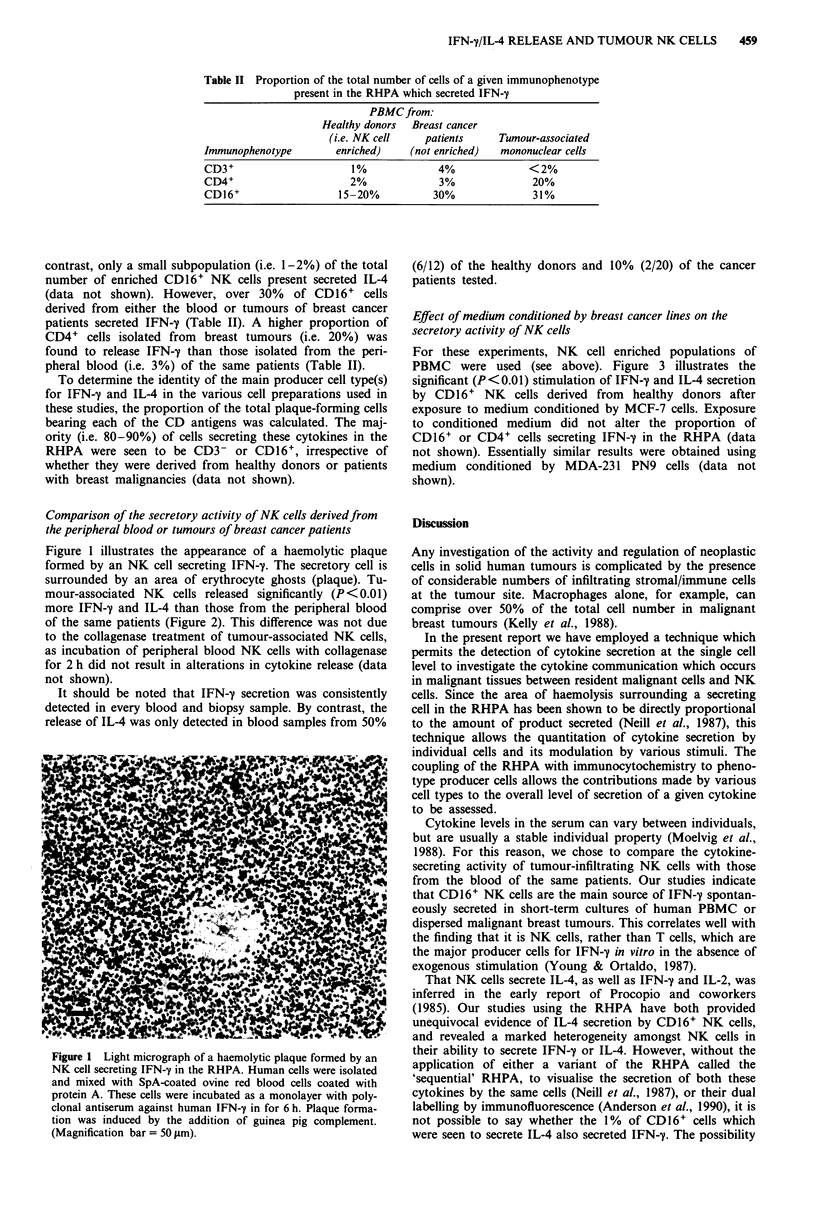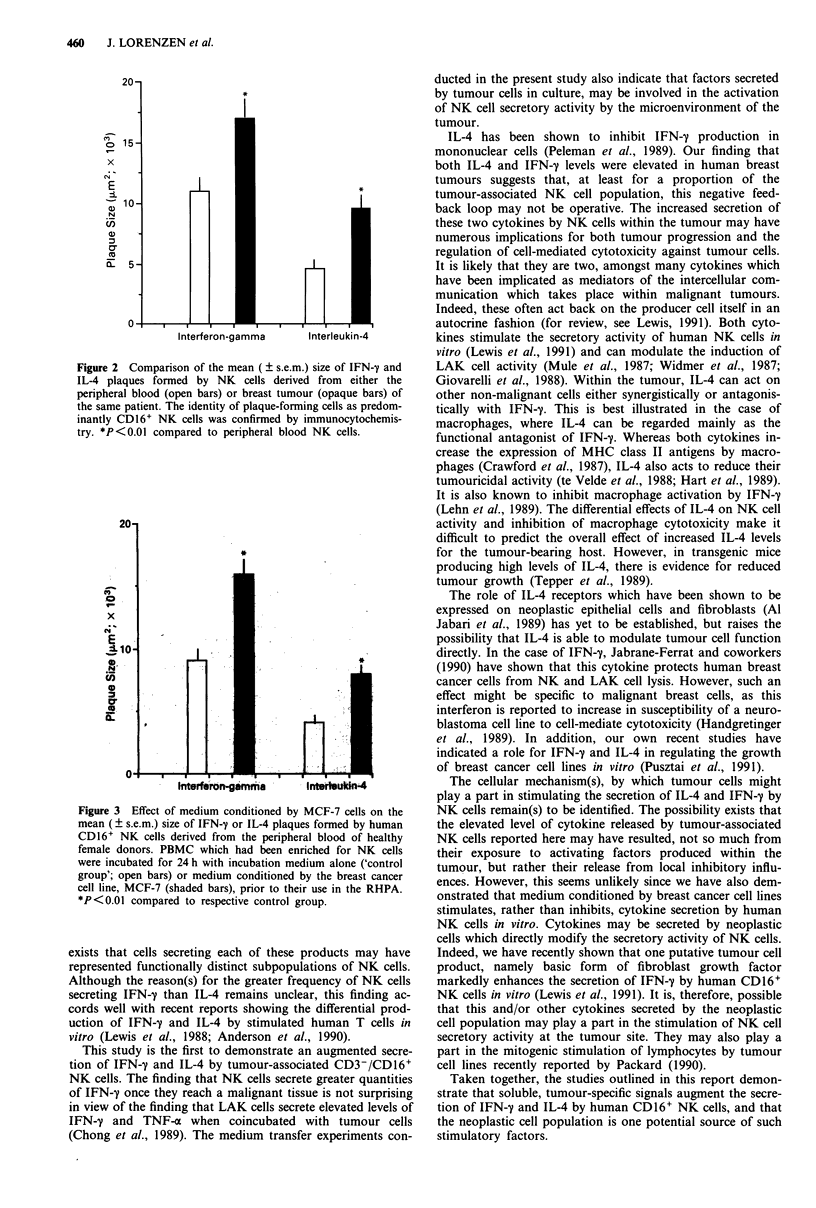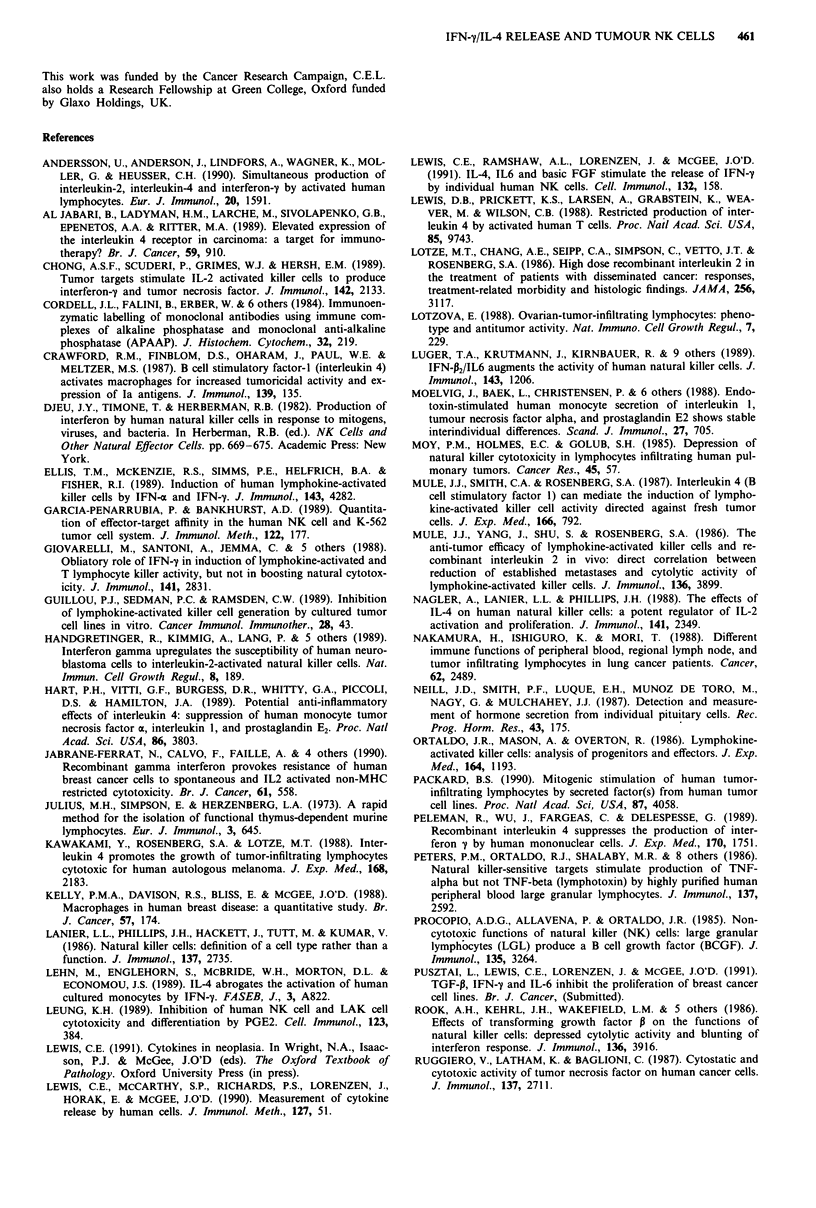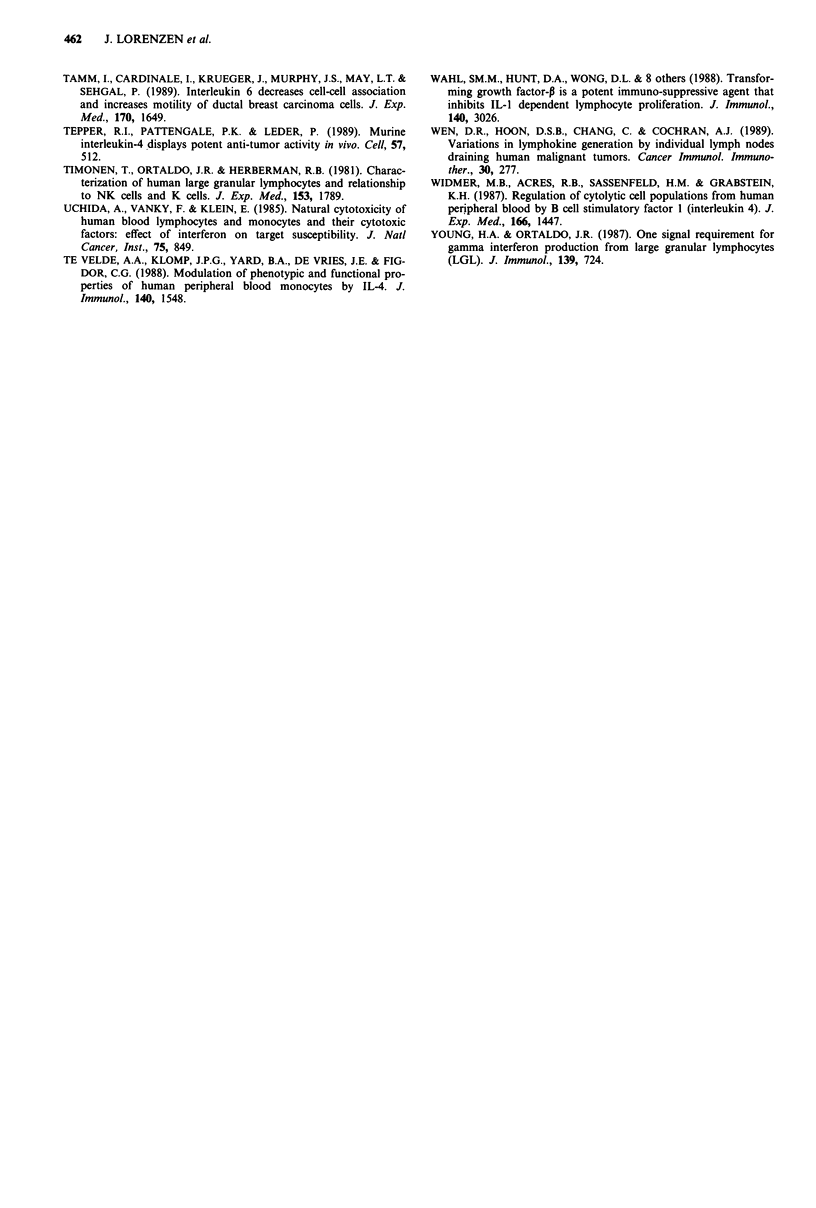# Human tumour-associated NK cells secrete increased amounts of interferon-gamma and interleukin-4.

**DOI:** 10.1038/bjc.1991.331

**Published:** 1991-09

**Authors:** J. Lorenzen, C. E. Lewis, D. McCracken, E. Horak, M. Greenall, J. O. McGee

**Affiliations:** Nuffield Department of Pathology and Bacteriology, University of Oxford, UK.

## Abstract

**Images:**


Br. J. Cancer (1991), 64, 457–462 © Macmillan Press Ltd., 1991

### Human tumour-associated NK cells secrete increased amounts of Interferon-γ and Interleukin-4

J. Lorenzen[1], C.E. Lewis[1], D. McCracken[1], E. Horak[1], M. Greenall[2] & J.O'D. McGee[1]

[1]*Nuffield Department of Pathology and Bacteriology, University of Oxford and* [2]*Department of Surgery, John Radcliffe Hospital, Oxford OX3 9DU, UK.*

**Summary** Numerous interactions between malignant and stromal/inflammatory cells take place within solid human tumours, which are mediated, in part, by the release of signalling proteins called cytokines. In the present study, we have compared the secretion of two important immunomodulatory cytokines, IFN-γ and IL-4 by individual, immunophenotyped NK cells freshly isolated from either malignant tumour biopsies, or peripheral blood samples from patients with ductal invasive breast cancer. Due to the marked heterogeneity amongst cells isolated from these clinical samples, we have employed a technique called the reverse haemolytic plaque assay to identify and enumerate cytokine-secreting cells at the single cell level. Our data indicate that NK cells isolated directly from the tumour site secrete more IFN-γ and IL-4 than NK cells from the blood of the same patients. However, a greater proportion of $CD16^+$ cells from both sources in breast cancer patients secreted IFN-γ than of those from the blood of healthy donors. We also show that factors secreted by the human breast cell lines, MCF-7 and MDA-231 PN9, were able to mimic the stimulatory influence of the tumour microenvironment on secretory activity of NK cells.

Natural killer (NK) cells can be defined on the basis of their ability to lyse certain tumour cells in a manner which is not dependent upon the expression of MHC molecules. In man, such 'natural' cytotoxicity is displayed mainly by $CD3^-$/$CD16^+$ cells with the morphology of large granular lymphocytes (LGL) (Timonene *et al.*, 1981; Ortaldo *et al.*, 1986; Lanier *et al.*, 1986). NK cells can also be stimulated by IL-2 or IFN-γ to express a broader range of cytotoxicity, known as lymphokine activated killer (LAK) activity. Their ability to lyse tumour cells *in vitro* has led to the experimental use of NK and LAK cells in the immunotherapy of some types of human cancer. This has occasionally led to tumour regression and/or improved clinical prognosis in advanced renal cell cancers and melanomas (Lotze *et al.*, 1986; Mule *et al.*, 1986).

However, there is also evidence to suggest that the cytotoxicity of NK cells towards tumour cells may actually be markedly reduced once they infiltrate the microenvironment of a malignant tumour (Moy *et al.*, 1985; Lotzova, 1988; Nakamura *et al.*, 1988). Indeed, malignant tissues release factors which suppress various immune functions in draining lymph nodes (Wen *et al.*, 1989), and cultured tumour cells have been shown to inhibit the generation of LAK cells *in vitro* (Guillou *et al.*, 1989). That factors produced by the malignant cell population itself may mediate, at least in part, this immunosuppression is given credance by the finding that factors known to be produced by human cancer cells, such as TGF-β or prostaglandin $E_2$, markedly inhibit the cytotoxicity of NK and LAK cells *in vitro* (Rook *et al.*, 1986; Wahl *et al.*, 1988; Leung, 1989; Luger *et al.*, 1989).

NK cells secrete a range of cytokines, including IFN-γ, IL-2, (Djeu *et al.*, 1982) and TNF-α (Peters *et al.*, 1986) which are capable of directly lysing neoplastic cells in a time dependent manner (Uchida *et al.*, 1985; Ruggerio *et al.*, 1987). Recently, interleukins 4 and 6, which are also NK cell products (Tamm *et al.*, 1989) have been shown to alter the proliferation and metastatic potential of malignant cells *in vitro* (Pusztai *et al.*, 1991). Taken together, these findings suggest that NK cells may contribute a number of cytokines to the complex cytokine network which exists within the microenvironment of solid human tumours (Lewis, 1991), and which collectively regulates the activity of both resident neoplastic cells and immune cells infiltrating such tissues.

A sensitive immunoassay called the reverse haemolytic plaque assay (RHPA) has recently been introduced for the measurement of cytokine secretion by individual, immunoidentified human cells (Lewis *et al.*, 1990). In the present report, the RHPA has been used to investigate the influence of the tumour microenvironment on the release of IFN-γ and IL-4 by individual human $CD16^+$ NK cells. To do this, the secretory activity of NK cells derived from both tumour and the peripheral blood samples of patients with ductal invasive breast carcinoma were compared. In addition, experiments were undertaken to investigate the effects of soluble mediators released by the breast adenocarcinoma cell lines, MCF-7 and MDA-231 PN9, on the secretion of cytokines by NK cells *in vitro*.

### Materials and methods

### *Isolation of mononuclear cells from the peripheral blood of cancer patients*

Peripheral blood samples (10 ml) were obtained from patients undergoing mastectomy (prior to surgery). Peripheral blood mononuclear cells (PBMC) were isolated using Ficoll-Hypaque density gradients. Briefly, this involved diluting whole blood samples 1:1 (v/v) in calcium and magnesium-free Hank's buffered salt solution (HBSS). The resultant cell suspensions were then washed three times before being carefully layered onto Ficoll-Hypaque cushions. After centrifugation for 25 min at 450 *g*, cells in the lymphocyte layer were collected and washed by centrifugation/resuspension three times in HBSS. This procedure routinely yielded $1-5 \times 10^6$ cells $ml^{-1}$.

### *Enrichment of NK cells from the peripheral blood of healthy donors*

Human NK cells were isolated from 1,000 ml buffy coat preparations from five healthy female donors. PBMC were isolated using Ficoll-Hypaque density gradients as outlined above. The resulting cell pellet was resuspended in complete culture medium (see below) and incubated in large plastic culture dishes for 60 min at 37°C to remove plastic-adherent monocytes. Non-adherent cells were harvested and then incubated in nylon wool columns for 30 min at 37°C to

Correspondence: C.E. Lewis.
Received 19 February 1991; and in revised form 5 April 1991.

remove B cells (Julius et al., 1973). T cells were removed by the negative panning technique (Garcia-Penarrubia & Bankhurst, 1989). Briefly, this involved incubating cells ($5 \times 10^6$ cells $ml^{-1}$) with monoclonal antibodies ($10\,\mu l\ ml^{-1}$) specific for the CD3, CD4 and CD8 surface antigens on T cells (Dako, High Wycombe, UK) for 30 min at 4°C. The cell suspension was then layered onto polystyrene Petri dishes (Sterilin, UK), previously coated with goat anti-mouse IgG, for 60 min at 4°C. Non-adherent lymphoid cells (i.e. mainly NK cells) were carefully aspirated from each dish and used for subsequent experiments.

Unfortunately, experiments using breast cancer patient's PBMC enriched for NK cells could not be performed as the negative panning procedure requires 500–1,000 ml of whole blood. For obvious clinical reasons, the removal of this quantity of blood from each breast cancer patient was not feasible.

*Enzymatic dispersion of cells in malignant breast biopsies*

Mastectomy resections for breast cancer were collected immediately after surgery. Each tumour was dissected out and a portion removed for histopathological diagnosis. At the same time, a small ($1\ cm^3$) piece of tissue containing tumour was also removed and digested with 0.1% collagenase type IV (Sigma, Dorset, UK) in RPMI 1640 medium for 2 h at 22°C. Enzymatically dispersed cells were removed, suspended in 10 ml of HBSS and centrifuged at 444 *g* for 15 min. The supernatant was discarded and this step repeated four times in order to remove contaminating enzyme. This procedure routinely yielded $1-20 \times 10^6$ cells $ml^{-1}$. Monocytes were removed by adherence to plastic culture wells during a 60 min incubation. As a control, peripheral blood mononuclear cells from each of the cancer patients were subjected to similar collagenase treatment for 2 h and their secretory activity assessed in the RHPA.

*Culture conditions and preparation of tumour cell supernatants*

Cultures of MCF-7 or MDA-231-PN-9 cells were maintained in RPMI 1640 medium supplemented with 2 mM of glutamine, 10% foetal calf serum, 100 IU $ml^{-1}$ penicillin G and 100 $\mu g\ ml^{-1}$ streptomycin (complete culture medium) at 37°C in air/5% $CO_2$. All culture reagents used were obtained from Gibco, Paisley, UK. The human breast cancer cell lines, MCF-7 and MDA-231 PN-9, were propagated in complete culture to medium to near (>90%) confluence. Cells were incubated with fresh medium for 24 h and the medium then removed and centrifuged at 5,000 *g* for 20 min. The supernatant was filtered (pore size 0.2 μm) and used immediately as conditioned medium in subsequent medium-transfer experiments. Small aliquots of each batch of conditioned medium were transferred to glass slides and stained with haemotoxylin/eosin to check for the absence of any contaminating tumour cells.

*Reverse haemolytic plaque assay*

The secretion of IFN-γ and IL-4 was measured using the RHPA as previously described for cytokines (Lewis et al., 1990). Mononuclear human cells were suspended in RPMI 1640 medium supplemented with 0.1% bovine serum albumin, 2 mM glutamine and antibiotics (assay medium) and then mixed with ovine red blood cells (Serotec, Kidlington, UK) to which staphylococcal protein A (Sigma Chemicals, Dorset, UK) had been coupled using the chromium chloride method. This cell suspension was allowed to settle in Cunningham chambers for 45 min at 37°C in air/5% $CO_2$. Non-attached cells were removed with prewarmed assay medium and the chambers filled with a 1:50 dilution of polyclonal rabbit antisera to either native human IFN-γ or recombinant human IL-4 (Genzyme, USA). After incubation for 6 h, plaque formation was induced by infusion into the chambers of a 1:50 dilution of guinea pig complement in assay medium. The cell monolayers were then fixed with 2% glutaraldehyde (v/v) in phosphate buffered saline and stored at 4°C for subsequent analysis. All treatments were repeated on 10 separate slides in each assay.

*Immunocytochemistry*

Mononuclear cells were phenotyped by immunocytochemistry with monoclonal antibodies against CD3, CD4, CD8 (Dako, UK) and CD16 antigens (Becton Dickinson, UK). Slides were incubated with the monoclonal antibodies in Tris-buffered saline (TBS) with 5% low fat milk protein ('Marvel', Premier Brands, Birmingham, UK) at optimal dilutions for 4 h at 22°C. After several washes in TBS, bound antibodies were detected by the APAAP technique (Cordell et al., 1984) using fast red as chromogen. Nuclei were lightly counterstained with haematoxylin.

*Morphometry and statistics*

The area of the plaques formed in the RHPA was determined using a WILD M-20 microscope to which a drawing device had been attached. This allowed the superimposition of the microscopic image onto the screen of an Apple Macintosh computer. Plaques were traced manually with a cursor and areas calculated by a program developed by Dr J. Lorenzen in this Department. For IFN-γ secretion, the size of the first 20 randomly selected haemolytic plaques was measured on each of 5–10 replicate slides for each treatment (i.e. minimum of 100 plaques). For IL-4 secretion, all plaques on replicate slides were measured (as there was invariably fewer of the latter). To rule out subjective bias, all plaque sampling was performed 'blind' without prior knowledge of the source of NK cells. Statistical analysis of the data was performed using the Mann-Whitney U test. Although data from single, representative experiments have been illustrated in this report, essentially similar results were obtained in five identical assays using blood or tumour samples from at least five separate individuals.

### Results

*Immunocytochemical labelling of cell preparations used in the RHPA*

Table I indicates the higher proportions of cells expressing T (CD3, CD4 and CD8) and NK cell (CD16) antigens in PBMC isolated from breast cancer patients (after Ficoll-hypaque purification) than in cell preparations directly isolated from enzymatically-dispersed breast tumours. However, a slightly higher frequency of $CD16^+$ cells were seen in dispersed tumour-associated mononuclear cells.

Negative panning of PBMC from healthy donors reproducibly enriched the proportion of $CD16^+$ cells to >65%, and depleted cells bearing the CD3, CD4 and CD8 antigens to <5% (data not shown). Fifteen to twenty per cent of $CD16^+$ NK cells from healthy donors enriched in this way secreted IFN-γ, whereas fewer than 2% of $CD3^+$ or $CD4^+$ cells secreted this cytokine in the RHPA (Table II). By

**Table I** Frequency of cells bearing various CD antigens in cell preparations used in the RHPA

| *Immunophenotype* | *PMBC* | *Tumour-associated mononuclear cells* |
|---|---|---|
| $CD3^+$ | 64% | 18% |
| $CD4^+$ | 26% | 16% |
| $CD8^+$ | 14% | 2% |
| $CD16^+$ | 10% | 17% |

Peripheral blood mononuclear cells (PBMC) were isolated from the peripheral blood of a breast cancer patient using a Ficoll-Hypaque gradient. Tumour-associated mononuclear cells were isolated by enzymatic dispersion of the malignant breast biopsy from the same patient.

**Table II** Proportion of the total number of cells of a given immunophenotype present in the RHPA which secreted IFN-γ

| | *PBMC from:* | | |
|---|---|---|---|
| *Immunophenotype* | *Healthy donors (i.e. NK cell enriched)* | *Breast cancer patients (not enriched)* | *Tumour-associated mononuclear cells* |
| $CD3^+$ | 1% | 4% | <2% |
| $CD4^+$ | 2% | 3% | 20% |
| $CD16^+$ | 15–20% | 30% | 31% |

contrast, only a small subpopulation (i.e. 1–2%) of the total number of enriched $CD16^+$ NK cells present secreted IL-4 (data not shown). However, over 30% of $CD16^+$ cells derived from either the blood or tumours of breast cancer patients secreted IFN-γ (Table II). A higher proportion of $CD4^+$ cells isolated from breast tumours (i.e. 20%) was found to release IFN-γ than those isolated from the peripheral blood (i.e. 3%) of the same patients (Table II).

To determine the identity of the main producer cell type(s) for IFN-γ and IL-4 in the various cell preparations used in these studies, the proportion of the total plaque-forming cells bearing each of the CD antigens was calculated. The majority (i.e. 80–90%) of cells secreting these cytokines in the RHPA were seen to be $CD3^-$ or $CD16^+$, irrespective of whether they were derived from healthy donors or patients with breast malignancies (data not shown).

*Comparison of the secretory activity of NK cells derived from the peripheral blood or tumours of breast cancer patients*

Figure 1 illustrates the appearance of a haemolytic plaque formed by an NK cell secreting IFN-γ. The secretory cell is surrounded by an area of erythrocyte ghosts (plaque). Tumour-associated NK cells released significantly ($P<0.01$) more IFN-γ and IL-4 than those from the peripheral blood of the same patients (Figure 2). This difference was not due to the collagenase treatment of tumour-associated NK cells, as incubation of peripheral blood NK cells with collagenase for 2 h did not result in alterations in cytokine release (data not shown).

It should be noted that IFN-γ secretion was consistently detected in every blood and biopsy sample. By contrast, the release of IL-4 was only detected in blood samples from 50%

**Figure 1** Light micrograph of a haemolytic plaque formed by an NK cell secreting IFN-γ in the RHPA. Human cells were isolated and mixed with SpA-coated ovine red blood cells coated with protein A. These cells were incubated as a monolayer with polyclonal antiserum against human IFN-γ in for 6 h. Plaque formation was induced by the addition of guinea pig complement. (Magnification bar = 50 μm).

(6/12) of the healthy donors and 10% (2/20) of the cancer patients tested.

*Effect of medium conditioned by breast cancer lines on the secretory activity of NK cells*

For these experiments, NK cell enriched populations of PBMC were used (see above). Figure 3 illustrates the significant ($P<0.01$) stimulation of IFN-γ and IL-4 secretion by $CD16^+$ NK cells derived from healthy donors after exposure to medium conditioned by MCF-7 cells. Exposure to conditioned medium did not alter the proportion of $CD16^+$ or $CD4^+$ cells secreting IFN-γ in the RHPA (data not shown). Essentially similar results were obtained using medium conditioned by MDA-231 PN9 cells (data not shown).

### Discussion

Any investigation of the activity and regulation of neoplastic cells in solid human tumours is complicated by the presence of considerable numbers of infiltrating stromal/immune cells at the tumour site. Macrophages alone, for example, can comprise over 50% of the total cell number in malignant breast tumours (Kelly *et al.*, 1988).

In the present report we have employed a technique which permits the detection of cytokine secretion at the single cell level to investigate the cytokine communication which occurs in malignant tissues between resident malignant cells and NK cells. Since the area of haemolysis surrounding a secreting cell in the RHPA has been shown to be directly proportional to the amount of product secreted (Neill *et al.*, 1987), this technique allows the quantitation of cytokine secretion by individual cells and its modulation by various stimuli. The coupling of the RHPA with immunocytochemistry to phenotype producer cells allows the contributions made by various cell types to the overall level of secretion of a given cytokine to be assessed.

Cytokine levels in the serum can vary between individuals, but are usually a stable individual property (Moelvig *et al.*, 1988). For this reason, we chose to compare the cytokine-secreting activity of tumour-infiltrating NK cells with those from the blood of the same patients. Our studies indicate that $CD16^+$ NK cells are the main source of IFN-γ spontaneously secreted in short-term cultures of human PBMC or dispersed malignant breast tumours. This correlates well with the finding that it is NK cells, rather than T cells, which are the major producer cells for IFN-γ *in vitro* in the absence of exogenous stimulation (Young & Ortaldo, 1987).

That NK cells secrete IL-4, as well as IFN-γ and IL-2, was inferred in the early report of Procopio and coworkers (1985). Our studies using the RHPA have both provided unequivocal evidence of IL-4 secretion by $CD16^+$ NK cells, and revealed a marked heterogeneity amongst NK cells in their ability to secrete IFN-γ or IL-4. However, without the application of either a variant of the RHPA called the 'sequential' RHPA, to visualise the secretion of both these cytokines by the same cells (Neill *et al.*, 1987), or their dual labelling by immunofluorescence (Anderson *et al.*, 1990), it is not possible to say whether the 1% of $CD16^+$ cells which were seen to secrete IL-4 also secreted IFN-γ. The possibility

**Figure 2** Comparison of the mean (± s.e.m.) size of IFN-γ and IL-4 plaques formed by NK cells derived from either the peripheral blood (open bars) or breast tumour (opaque bars) of the same patient. The identity of plaque-forming cells as predominantly $CD16^+$ NK cells was confirmed by immunocytochemistry. $*P<0.01$ compared to peripheral blood NK cells.

**Figure 3** Effect of medium conditioned by MCF-7 cells on the mean (± s.e.m.) size of IFN-γ or IL-4 plaques formed by human $CD16^+$ NK cells derived from the peripheral blood of healthy female donors. PBMC which had been enriched for NK cells were incubated for 24 h with incubation medium alone ('control group'; open bars) or medium conditioned by the breast cancer cell line, MCF-7 (shaded bars), prior to their use in the RHPA. $*P<0.01$ compared to respective control group.

exists that cells secreting each of these products may have represented functionally distinct subpopulations of NK cells. Although the reason(s) for the greater frequency of NK cells secreting IFN-γ than IL-4 remains unclear, this finding accords well with recent reports showing the differential production of IFN-γ and IL-4 by stimulated human T cells *in vitro* (Lewis *et al.*, 1988; Anderson *et al.*, 1990).

This study is the first to demonstrate an augmented secretion of IFN-γ and IL-4 by tumour-associated $CD3^-/CD16^+$ NK cells. The finding that NK cells secrete greater quantities of IFN-γ once they reach a malignant tissue is not surprising in view of the finding that LAK cells secrete elevated levels of IFN-γ and TNF-α when coincubated with tumour cells (Chong *et al.*, 1989). The medium transfer experiments conducted in the present study also indicate that factors secreted by tumour cells in culture, may be involved in the activation of NK cell secretory activity by the microenvironment of the tumour.

IL-4 has been shown to inhibit IFN-γ production in mononuclear cells (Peleman *et al.*, 1989). Our finding that both IL-4 and IFN-γ levels were elevated in human breast tumours suggests that, at least for a proportion of the tumour-associated NK cell population, this negative feedback loop may not be operative. The increased secretion of these two cytokines by NK cells within the tumour may have numerous implications for both tumour progression and the regulation of cell-mediated cytotoxicity against tumour cells. It is likely that they are two, amongst many cytokines which have been implicated as mediators of the intercellular communication which takes place within malignant tumours. Indeed, these often act back on the producer cell itself in an autocrine fashion (for review, see Lewis, 1991). Both cytokines stimulate the secretory activity of human NK cells *in vitro* (Lewis *et al.*, 1991) and can modulate the induction of LAK cell activity (Mule *et al.*, 1987; Widmer *et al.*, 1987; Giovarelli *et al.*, 1988). Within the tumour, IL-4 can act on other non-malignant cells either synergistically or antagonistically with IFN-γ. This is best illustrated in the case of macrophages, where IL-4 can be regarded mainly as the functional antagonist of IFN-γ. Whereas both cytokines increase the expression of MHC class II antigens by macrophages (Crawford *et al.*, 1987), IL-4 also acts to reduce their tumouricidal activity (te Velde *et al.*, 1988; Hart *et al.*, 1989). It is also known to inhibit macrophage activation by IFN-γ (Lehn *et al.*, 1989). The differential effects of IL-4 on NK cell activity and inhibition of macrophage cytotoxicity make it difficult to predict the overall effect of increased IL-4 levels for the tumour-bearing host. However, in transgenic mice producing high levels of IL-4, there is evidence for reduced tumour growth (Tepper *et al.*, 1989).

The role of IL-4 receptors which have been shown to be expressed on neoplastic epithelial cells and fibroblasts (Al Jabari *et al.*, 1989) has yet to be established, but raises the possibility that IL-4 is able to modulate tumour cell function directly. In the case of IFN-γ, Jabrane-Ferrat and coworkers (1990) have shown that this cytokine protects human breast cancer cells from NK and LAK cell lysis. However, such an effect might be specific to malignant breast cells, as this interferon is reported to increase in susceptibility of a neuroblastoma cell line to cell-mediate cytotoxicity (Handgretinger *et al.*, 1989). In addition, our own recent studies have indicated a role for IFN-γ and IL-4 in regulating the growth of breast cancer cell lines *in vitro* (Pusztai *et al.*, 1991).

The cellular mechanism(s), by which tumour cells might play a part in stimulating the secretion of IL-4 and IFN-γ by NK cells remain(s) to be identified. The possibility exists that the elevated level of cytokine released by tumour-associated NK cells reported here may have resulted, not so much from their exposure to activating factors produced within the tumour, but rather their release from local inhibitory influences. However, this seems unlikely since we have also demonstrated that medium conditioned by breast cancer cell lines stimulates, rather than inhibits, cytokine secretion by human NK cells *in vitro*. Cytokines may be secreted by neoplastic cells which directly modify the secretory activity of NK cells. Indeed, we have recently shown that one putative tumour cell product, namely basic form of fibroblast growth factor markedly enhances the secretion of IFN-γ by human $CD16^+$ NK cells *in vitro* (Lewis *et al.*, 1991). It is, therefore, possible that this and/or other cytokines secreted by the neoplastic cell population may play a part in the stimulation of NK cell secretory activity at the tumour site. They may also play a part in the mitogenic stimulation of lymphocytes by tumour cell lines recently reported by Packard (1990).

Taken together, the studies outlined in this report demonstrate that soluble, tumour-specific signals augment the secretion of IFN-γ and IL-4 by human $CD16^+$ NK cells, and that the neoplastic cell population is one potential source of such stimulatory factors.

This work was funded by the Cancer Research Campaign, C.E.L. also holds a Research Fellowship at Green College, Oxford funded by Glaxo Holdings, UK.

### References

ANDERSSON, U., ANDERSON, J., LINDFORS, A., WAGNER, K., MOLLER, G. & HEUSSER, C.H. (1990). Simultaneous production of interleukin-2, interleukin-4 and interferon-γ by activated human lymphocytes. *Eur. J. Immunol.*, **20**, 1591.

AL JABARI, B., LADYMAN, H.M., LARCHE, M., SIVOLAPENKO, G.B., EPENETOS, A.A. & RITTER, M.A. (1989). Elevated expression of the interleukin 4 receptor in carcinoma: a target for immunotherapy? *Br. J. Cancer*, **59**, 910.

CHONG, A.S.F., SCUDERI, P., GRIMES, W.J. & HERSH, E.M. (1989). Tumor targets stimulate IL-2 activated killer cells to produce interferon-γ and tumor necrosis factor. *J. Immunol.*, **142**, 2133.

CORDELL, J.L., FALINI, B., ERBER, W. & 6 others (1984). Immunoenzymatic labelling of monoclonal antibodies using immune complexes of alkaline phosphatase and monoclonal anti-alkaline phosphatase (APAAP). *J. Histochem. Cytochem.*, **32**, 219.

CRAWFORD, R.M., FINBLOM, D.S., OHARAM, J., PAUL, W.E. & MELTZER, M.S. (1987). B cell stimulatory factor-1 (interleukin 4) activates macrophages for increased tumoricidal activity and expression of Ia antigens. *J. Immunol.*, **139**, 135.

DJEU, J.Y., TIMONE, T. & HERBERMAN, R.B. (1982). Production of interferon by human natural killer cells in response to mitogens, viruses, and bacteria. In Herberman, R.B. (ed.). *NK Cells and Other Natural Effector Cells*. pp. 669–675. Academic Press: New York.

ELLIS, T.M., MCKENZIE, R.S., SIMMS, P.E., HELFRICH, B.A. & FISHER, R.I. (1989). Induction of human lymphokine-activated killer cells by IFN-α and IFN-γ. *J. Immunol.*, **143**, 4282.

GARCIA-PENARRUBIA, P. & BANKHURST, A.D. (1989). Quantitation of effector-target affinity in the human NK cell and K-562 tumor cell system. *J. Immunol. Meth.*, **122**, 177.

GIOVARELLI, M., SANTONI, A., JEMMA, C. & 5 others (1988). Obliatory role of IFN-γ in induction of lymphokine-activated and T lymphocyte killer activity, but not in boosting natural cytotoxicity. *J. Immunol.*, **141**, 2831.

GUILLOU, P.J., SEDMAN, P.C. & RAMSDEN, C.W. (1989). Inhibition of lymphokine-activated killer cell generation by cultured tumor cell lines in vitro. *Cancer Immunol. Immunother.*, **28**, 43.

HANDGRETINGER, R., KIMMIG, A., LANG, P. & 5 others (1989). Interferon gamma upregulates the susceptibility of human neuroblastoma cells to interleukin-2-activated natural killer cells. *Nat. Immun. Cell Growth Regul.*, **8**, 189.

HART, P.H., VITTI, G.F., BURGESS, D.R., WHITTY, G.A., PICCOLI, D.S. & HAMILTON, J.A. (1989). Potential anti-inflammatory effects of interleukin 4: suppression of human monocyte tumor necrosis factor α, interleukin 1, and prostaglandin $E_2$. *Proc. Natl Acad. Sci. USA*, **86**, 3803.

JABRANE-FERRAT, N., CALVO, F., FAILLE, A. & 4 others (1990). Recombinant gamma interferon provokes resistance of human breast cancer cells to spontaneous and IL2 activated non-MHC restricted cytotoxicity. *Br. J. Cancer*, **61**, 558.

JULIUS, M.H., SIMPSON, E. & HERZENBERG, L.A. (1973). A rapid method for the isolation of functional thymus-dependent murine lymphocytes. *Eur. J. Immunol.*, **3**, 645.

KAWAKAMI, Y., ROSENBERG, S.A. & LOTZE, M.T. (1988). Interleukin 4 promotes the growth of tumor-infiltrating lymphocytes cytotoxic for human autologous melanoma. *J. Exp. Med.*, **168**, 2183.

KELLY, P.M.A., DAVISON, R.S., BLISS, E. & MCGEE, J.O'D. (1988). Macrophages in human breast disease: a quantitative study. *Br. J. Cancer*, **57**, 174.

LANIER, L.L., PHILLIPS, J.H., HACKETT, J., TUTT, M. & KUMAR, V. (1986). Natural killer cells: definition of a cell type rather than a function. *J. Immunol.*, **137**, 2735.

LEHN, M., ENGLEHORN, S., MCBRIDE, W.H., MORTON, D.L. & ECONOMOU, J.S. (1989). IL-4 abrogates the activation of human cultured monocytes by IFN-γ. *FASEB, J.*, **3**, A822.

LEUNG, K.H. (1989). Inhibition of human NK cell and LAK cell cytotoxicity and differentiation by PGE2. *Cell. Immunol.*, **123**, 384.

LEWIS, C.E. (1991). Cytokines in neoplasia. In Wright, N.A., Isaacson, P.J. & McGee, J.O'D (eds). *The Oxford Textbook of Pathology*. Oxford University Press (in press).

LEWIS, C.E., MCCARTHY, S.P., RICHARDS, P.S., LORENZEN, J., HORAK, E. & MCGEE, J.O'D. (1990). Measurement of cytokine release by human cells. *J. Immunol. Meth.*, **127**, 51.

LEWIS, C.E., RAMSHAW, A.L., LORENZEN, J. & MCGEE, J.O'D. (1991). IL-4, IL6 and basic FGF stimulate the release of IFN-γ by individual human NK cells. *Cell. Immunol.*, **132**, 158.

LEWIS, D.B., PRICKETT, K.S., LARSEN, A., GRABSTEIN, K., WEAVER, M. & WILSON, C.B. (1988). Restricted production of interleukin 4 by activated human T cells. *Proc. Natl Acad. Sci. USA*, **85**, 9743.

LOTZE, M.T., CHANG, A.E., SEIPP, C.A., SIMPSON, C., VETTO, J.T. & ROSENBERG, S.A. (1986). High dose recombinant interleukin 2 in the treatment of patients with disseminated cancer: responses, treatment-related morbidity and histologic findings. *JAMA*, **256**, 3117.

LOTZOVA, E. (1988). Ovarian-tumor-infiltrating lymphocytes: phenotype and antitumor activity. *Nat. Immuno. Cell Growth Regul.*, **7**, 229.

LUGER, T.A., KRUTMANN, J., KIRNBAUER, R. & 9 others (1989). IFN-$\beta_2$/IL6 augments the activity of human natural killer cells. *J. Immunol.*, **143**, 1206.

MOELVIG, J., BAEK, L., CHRISTENSEN, P. & 6 others (1988). Endotoxin-stimulated human monocyte secretion of interleukin 1, tumour necrosis factor alpha, and prostaglandin E2 shows stable interindividual differences. *Scand. J. Immunol.*, **27**, 705.

MOY, P.M., HOLMES, E.C. & GOLUB, S.H. (1985). Depression of natural killer cytotoxicity in lymphocytes infiltrating human pulmonary tumors. *Cancer Res.*, **45**, 57.

MULE, J.J., SMITH, C.A. & ROSENBERG, S.A. (1987). Interleukin 4 (B cell stimulatory factor 1) can mediate the induction of lymphokine-activated killer cell activity directed against fresh tumor cells. *J. Exp. Med.*, **166**, 792.

MULE, J.J., YANG, J., SHU, S. & ROSENBERG, S.A. (1986). The anti-tumor efficacy of lymphokine-activated killer cells and recombinant interleukin 2 in vivo: direct correlation between reduction of established metastases and cytolytic activity of lymphokine-activated killer cells. *J. Immunol.*, **136**, 3899.

NAGLER, A., LANIER, L.L. & PHILLIPS, J.H. (1988). The effects of IL-4 on human natural killer cells: a potent regulator of IL-2 activation and proliferation. *J. Immunol.*, **141**, 2349.

NAKAMURA, H., ISHIGURO, K. & MORI, T. (1988). Different immune functions of peripheral blood, regional lymph node, and tumor infiltrating lymphocytes in lung cancer patients. *Cancer*, **62**, 2489.

NEILL, J.D., SMITH, P.F., LUQUE, E.H., MUNOZ DE TORO, M., NAGY, G. & MULCHAHEY, J.J. (1987). Detection and measurement of hormone secretion from individual pituitary cells. *Rec. Prog. Horm. Res.*, **43**, 175.

ORTALDO, J.R., MASON, A. & OVERTON, R. (1986). Lymphokine-activated killer cells: analysis of progenitors and effectors. *J. Exp. Med.*, **164**, 1193.

PACKARD, B.S. (1990). Mitogenic stimulation of human tumor-infiltrating lymphocytes by secreted factor(s) from human tumor cell lines. *Proc. Natl Acad. Sci, USA*, **87**, 4058.

PELEMAN, R., WU, J., FARGEAS, C. & DELESPESSE, G. (1989). Recombinant interleukin 4 suppresses the production of interferon γ by human mononuclear cells. *J. Exp. Med.*, **170**, 1751.

PETERS, P.M., ORTALDO, R.J., SHALABY, M.R. & 8 others (1986). Natural killer-sensitive targets stimulate production of TNF-alpha but not TNF-beta (lymphotoxin) by highly purified human peripheral blood large granular lymphocytes. *J. Immunol.*, **137**, 2592.

PROCOPIO, A.D.G., ALLAVENA, P. & ORTALDO, J.R. (1985). Non-cytotoxic functions of natural killer (NK) cells: large granular lymphocytes (LGL) produce a B cell growth factor (BCGF). *J. Immunol.*, **135**, 3264.

PUSZTAI, L., LEWIS, C.E., LORENZEN, J. & MCGEE, J.O'D. (1991). TGF-β, IFN-γ and IL-6 inhibit the proliferation of breast cancer cell lines. *Br. J. Cancer*, (Submitted).

ROOK, A.H., KEHRL, J.H., WAKEFIELD, L.M. & 5 others (1986). Effects of transforming growth factor β on the functions of natural killer cells: depressed cytolytic activity and blunting of interferon response. *J. Immunol.*, **136**, 3916.

RUGGIERO, V., LATHAM, K. & BAGLIONI, C. (1987). Cytostatic and cytotoxic activity of tumor necrosis factor on human cancer cells. *J. Immunol.*, **137**, 2711.

TAMM, I., CARDINALE, I., KRUEGER, J., MURPHY, J.S., MAY, L.T. & SEHGAL, P. (1989). Interleukin 6 decreases cell-cell association and increases motility of ductal breast carcinoma cells. *J. Exp. Med.*, **170**, 1649.

TEPPER, R.I., PATTENGALE, P.K. & LEDER, P. (1989). Murine interleukin-4 displays potent anti-tumor activity *in vivo*. *Cell*, **57**, 512.

TIMONEN, T., ORTALDO, J.R. & HERBERMAN, R.B. (1981). Characterization of human large granular lymphocytes and relationship to NK cells and K cells. *J. Exp. Med.*, **153**, 1789.

UCHIDA, A., VANKY, F. & KLEIN, E. (1985). Natural cytotoxicity of human blood lymphocytes and monocytes and their cytotoxic factors: effect of interferon on target susceptibility. *J. Natl Cancer, Inst.*, **75**, 849.

TE VELDE, A.A., KLOMP, J.P.G., YARD, B.A., DE VRIES, J.E. & FIGDOR, C.G. (1988). Modulation of phenotypic and functional properties of human peripheral blood monocytes by IL-4. *J. Immunol.*, **140**, 1548.

WAHL, SM.M., HUNT, D.A., WONG, D.L. & 8 others (1988). Transforming growth factor-β is a potent immuno-suppressive agent that inhibits IL-1 dependent lymphocyte proliferation. *J. Immunol.*, **140**, 3026.

WEN, D.R., HOON, D.S.B., CHANG, C. & COCHRAN, A.J. (1989). Variations in lymphokine generation by individual lymph nodes draining human malignant tumors. *Cancer Immunol. Immunother.*, **30**, 277.

WIDMER, M.B., ACRES, R.B., SASSENFELD, H.M. & GRABSTEIN, K.H. (1987). Regulation of cytolytic cell populations from human peripheral blood by B cell stimulatory factor 1 (interleukin 4). *J. Exp. Med.*, **166**, 1447.

YOUNG, H.A. & ORTALDO, J.R. (1987). One signal requirement for gamma interferon production from large granular lymphocytes (LGL). *J. Immunol.*, **139**, 724.